# Immobilization of *Yarrowia lipolytica* Lipase on Macroporous Resin Using Different Methods: Characterization of the Biocatalysts in Hydrolysis Reaction

**DOI:** 10.1155/2015/139179

**Published:** 2015-07-09

**Authors:** Jingjing Sun, Yiling Chen, Jun Sheng, Mi Sun

**Affiliations:** Laboratory of Enzyme Engineering, Yellow Sea Fisheries Research Institute, Chinese Academy of Fishery Sciences, Qingdao 266071, China

## Abstract

To improve the reusability and organic solvent tolerance of microbial lipase and expand the application of lipase (hydrolysis, esterification, and transesterification), we immobilized marine microbial lipase using different methods and determined the properties of immobilized lipases. Considering the activity and cost of immobilized lipase, the concentration of lipase was fixed at 2 mg/mL. The optimal temperature of immobilized lipases was 40°C and 5°C higher than free lipase. The activities of immobilized lipases were much higher than free lipase at alkaline pH (more than 50% at pH 12). The free lipase lost most activity (35.3%) and immobilized lipases retained more than 46.4% of their initial activity after 3 h heat treatment at 70°C. At alkaline pH, immobilized lipases were more stable than free lipase (more than 60% residue activity at pH 11 for 3 h). Immobilized lipases retained 80% of their activity after 5 cycles and increased enzyme activity (more than 108.7%) after 3 h treatment in tert-butanol. Immobilization of lipase which improved reusability of lipase and provided a chance to expand the application of marine microbial lipase in organic system expanded the application range of lipase to catalyze hydrolysis and esterification in harsh condition.

## 1. Introduction

Lipases (triacylglycerol hydrolases, E.C. 3.1.1.3) are enzymes that catalyze the hydrolysis of long chain triglycerides under physiological conditions [1–3]. They also can accept a broad range of substrates and catalyze interesterification [4–6] and esterification [3]. Lipases have some good characteristics such as high tolerance of the environment (pH, temperature, and organic solvents), require no cofactor for reaction, and are widely used on biological catalysis [7] such as biodiesel production [8–10], biopolymer synthesis [11], enantiopure synthesis of pharmaceuticals [12, 13], laundry formulations [14], and food chemistry [15].

Compared with chemical catalyst, the costs of separation and purification of enzyme are huge. Immobilization of enzyme can expand the application of biocatalysis technology and allow enzymes to easily separate from reaction mixture and recycle for next reaction. Furthermore, immobilization of enzyme can increase its thermal and chemical stability and resistance to extreme changes in conditions [16–18]. Lipases are very suitable to immobilization and propitious to industry application [19–23]. There are many immobilized methods for enzymes and the commonly used methods are physical adsorption, entrapment, cross-linkage, and covalent attachment [24]. Physical adsorption method can be achieved by mixing enzyme solution and solid support for a period of time and then removing unabsorbed enzyme by washing with buffer [25–29]. Advantages of adsorption method are easy operation, saving time, maintaining the enzyme activity, and being more economic. But it may not be strong enough and the enzymes probably fall off during washing and operation [30]. Entrapment method is trapped enzyme in insoluble beads or microspheres but this insoluble carrier may block the substrates in and the product out [31]. Cross-linkage method is to covalently bond enzymes together to create aggregates. The reaction may cover the active site of enzyme and block the substrate close to enzyme in space. Covalent attachment method is one of the most stabilized ways between enzyme and support. Covalent attachment method is a commonly used method for immobilization of enzymes. It can be implemented by connected enzyme and support by covalent linkage [32, 33]. There are couples of active sites in the enzyme, such as *ε*-amino group of lysine, thiol group of cysteine, carboxyl group of glutamate, and iminazole of histidine. These active groups are direct or through spacer arm attached to the functional groups of carrier (e.g., aldehyde group, carbodiimide, or maleinimide). However, the covalent attachment may reduce the activity of enzyme because of the inflexible linkage between enzyme and carrier [34].

**Figure 1:**
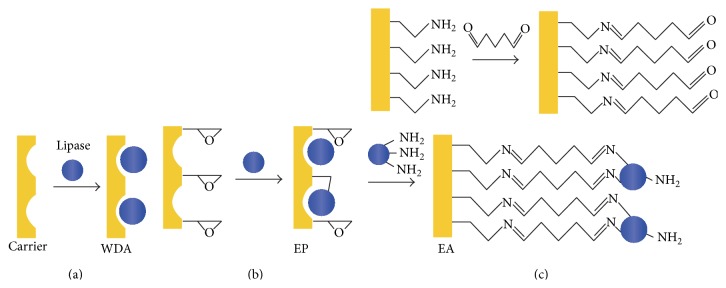
Principles of lipase immobilization: (a) macroporous resin (WDA); (b) support with epoxy group (EP); (c) support with amino group (EA).

In this work, our attention focused on the immobilization and biochemical characteristics of* Yarrowia lipolytica* lipase [35]. This lipase was cloned from* Yarrowia lipolytica* (Bohaisea-9145) and expressed in* Escherichia coli*. The optimal temperature and pH of the purified lipase to* p*-nitrophenyl laurate were 35°C and 8.5. The process of immobilization enzyme is complex, and single method may not be the appropriate one. So we compared three immobilization methods with different principles (Figure 1): macroporous resin (adsorption), support with epoxy group (adsorption and covalent attachment), and activated aminated support with glutaraldehyde (covalent attachment). The first immobilization method is based on the absorption of macroporous material. The epoxy-activated carrier can slowly multipointedly covalently attach to nucleophiles on the enzyme (e.g., amino, thiol, and hydroxyl groups) after adsorption [36, 37]. Activation of aminated carrier with glutaraldehyde could form Schiff bases with amine groups of enzyme and achieve multipoint covalent attachment [38–40].

## 2. Material and Methods

### 2.1. Materials

Lyophilized powder of marine* Yarrowia lipolytica* yeast lipase from our laboratory was used. WDA918 (macroporous acrylic acid series weakly acidic cation exchange resin) was purchased from Anhui Wandong Chemical Co., Ltd., China. LX-1000EP (epoxy resin) and LX-1000EA (amino resin) were acquired from Xi'an Lanxiao Technology Co., Ltd., China. Glutaraldehyde AR (50% in H_2_O) was purchased from Aladdin Industrial Inc. Tris(hydroxymethyl)aminomethane (Tris) was from Amresco Co.* P*-Nitrophenyl laurate was purchased from Sigma-Aldrich. Ultrapure water generated through a UNIQUE-S15 facility was used throughout the experiments.

### 2.2. Preparation of Lipase

*Yarrowia lipolytica* lipase was cloned in expression vector pET-21a(+) with a C-terminal His-tag and expressed in* Escherichia coli* BL21 (DE3). The transformants were cultured at 37°C in LB medium supplemented with 100 *μ*g/mL ampicillin until an OD600 of 0.4–0.6 was reached. IPTG was added to the medium at a final concentration of 0.5 mM. The cells were further cultured at 16°C for 12 h. The recombinant lipase was purified using a Ni-affinity column according to the manufacturer's protocol. The final product was freeze-dried for 12 h. The activity protein concentration (0.1 mg/mL) was 21.8 U/mL.

### 2.3. Immobilization of Lipase

To remove contaminants and keep proper pH of the surface, WDA918 and LX-1000EP (10 g) were separately treated by 40 mL 0.1 M pH 8.0 potassium phosphate buffer for 1 h and then freeze-dried for 8 h. Add lipase solution (40 mL 0.02 M pH 8 potassium phosphate buffer) to prepared WDA918 (10 g) or LX-1000EP (10 g). The mixture was at 25°C and shake speed was 180 rpm for 12 h. Then remove extra lipase and freeze-dry for 8 h. The immobilization products of WDA918 and LX-1000EP were named, respectively, WDA and EP.

LX-1000EA was treated by 40 mL 0.1 M pH 8 potassium phosphate buffer for 1 h and then freeze-dried for 8 h. Glutaraldehyde solution (2.5%, 0.02 M pH 8 potassium phosphate buffer) was added into the prepared LX-1000EA and reacted overnight. Then remove extra glutaraldehyde and freeze-dry for 8 h. Lipase solution (40 mL 0.02 M pH 8 potassium phosphate buffer) and the glutaraldehyde-treated LX-1000EA (10 g) were mixed together at 25°C and shake speed was 180 rpm for 12 h. Then remove extra lipase and freeze-dry for 8 h. The immobilization product of LX-1000EA was named EA.

### 2.4. Lipase Activity Assay

There are two solutions that were prepared before the lipase activity assay: solution A (83 mg of* p*-nitrophenyl laurate was dissolved in 25 mL of isopropanol) and solution B (100 mM potassium phosphate buffer; pH 8; 0.5% Triton X-100). Lipase (0.1 mL) solution or immobilized lipase was added to 1.5 mL solution B and 0.1 mL solution A. We mix up the mixture gently and the mixture was incubated for 8 min in a shaking water bath at 40°C. The inactivated lipase (0.1 mL) or inactivated immobilized lipase (heated at 100°C for 5 min) was used as control. Then, the reactions were terminated by putting the mixtures in ice and the OD (optical density) value at 410 nm of mixtures was read by spectrophotometer. One unit (U) of enzyme activity was defined as the amount of enzyme required for the liberation of 1 *μ*mol* p*-nitrophenol per minute under the assay conditions.

### 2.5. Effect of Lipase Concentration on Immobilization

Concentration gradients of lipase solutions (0.5, 1, 2, 4, and 6 mg/mL 0.02 M pH 8 potassium phosphate buffer) of 0.8 mL were incubated with 200 mg WDA918, LX-1000EP, or glutaraldehyde-treated LX-1000EA. Concentration gradients of lipase solutions were treated at the same condition as control. The enzyme activity of supernatants and control was assayed by the method which was described before. The relation between the percentages of bounding lipase and lipase concentration was calculated.

### 2.6. Optimal Temperature and pH of Immobilized Enzyme

The activity of immobilized enzyme (50 mg) or lipase (100 *μ*L) was assayed in potassium phosphate buffer (100 mM, pH 8) at different temperatures 20, 30, 35, 40, 50, 60, and 70°C. The activity of immobilized enzyme (50 mg) or lipase (100 *μ*L) was assayed in potassium phosphate buffer (pH 7, 8) and tris-HCl buffer (pH 9, 10, 11, and 12) at 40°C.

### 2.7. Temperature and pH Stability of Immobilized Enzyme

The thermal stability of immobilized enzyme or lipase was investigated by incubating the immobilized enzyme or lipase at 20, 30, 35, 40, 50, 60, and 70°C for 3 h in a water bath. The pH stability of immobilized enzyme or lipase was investigated by incubating the immobilized enzyme or lipase at different pH (7, 8, 9, 10, 11, and 12) for 3 h at room temperature.

### 2.8. Reusability of Immobilized Enzyme

Immobilized enzyme was assayed at 40°C for 8 min and washed three times using solution B. Repeat this process for 10 times and read the OD value of mixtures at 410 nm by spectrophotometer.

### 2.9. Effect of Organic Solvents on Immobilized Lipase Activity

The effect of various organic solvents (methanol, ethanol, acetone, chloroform, n-hexane, n-heptane, and tert-butanol) on immobilized enzyme was determined. The immobilized lipase was incubated with each of the selected solvents at room temperature for 3 h. Then the organic solvents were removed and the residual activity of immobilized lipases was determined.

## 3. Results and Discussion

### 3.1. Immobilization of Microbial Lipase

**Figure 2:**
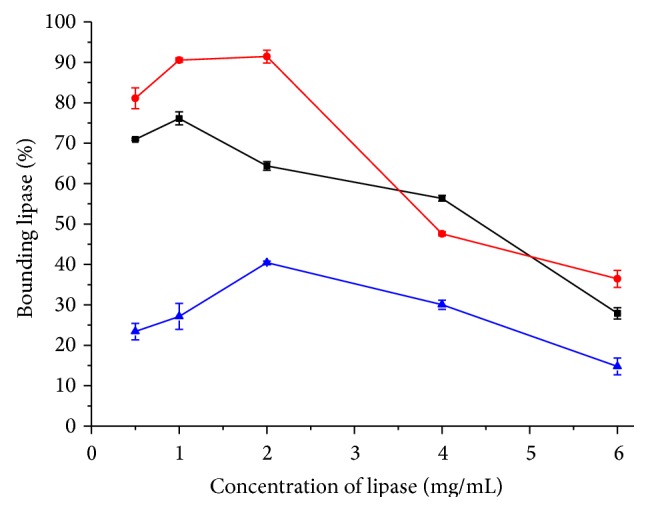
Effect of protein concentration on immobilization of lipase. This figure illustrated the relationship between the percentages of bounding lipase and lipase concentration. The color of curves represents the following: red: WDA; black: EP; blue: EA.

**Figure 3:**
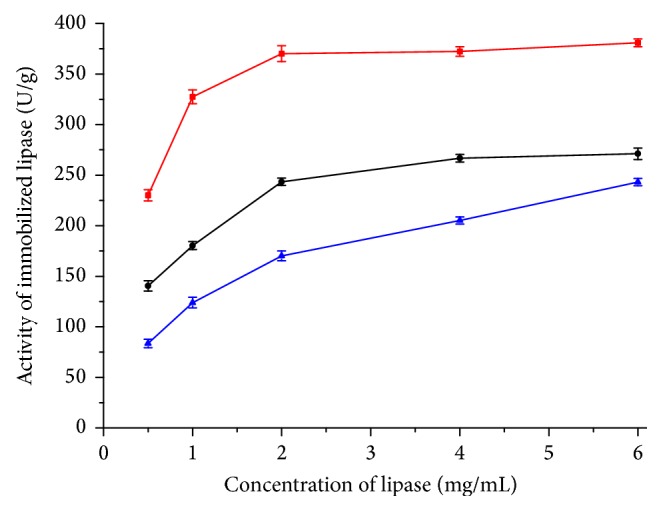
Effect of protein concentration on immobilization of lipase. This figure illustrated the relationship between the activity of immobilized lipase and lipase concentration. The color of curves represents the following: red: WDA; black: EP; blue: EA.

It is important to choose a proper immobilization method for enzyme. Bounding ability of carrier which is determined by residue activity of enzyme solution is one of the most important index immobilizations of enzyme. Different carriers perform different effects between protein concentration and bounding efficiency (Figure 2). When the protein concentration is between 0.5 mg/mL and 2 mg/mL, WDA performed with the highest bounding efficiency (more than 80%) compared to other carriers. With increasing protein concentration from 4 mg/mL to 6 mg/mL, the bounding efficiency of these three immobilization methods was almost at the same level (less than 60%) because of the carrier capacity. The bounding efficiency of EA was the lowest one because covalent attachment efficiency between lipase and carrier may limit the bounding opportunity of lipase. For activation of aminated carrier with glutaraldehyde (EA), the number of active groups on the surface of the carrier was fixed. When the concentration of protein was high, there were insufficient active groups for amino groups of lipase resulting in decreased modification rate [39, 41, 42]. Effect of protein concentration on immobilization of lipase is showed in Figure 3. When the protein concentration was more than 2 mg/mL, the activity of WDA and EP reached a plateau and EA increased steadily. Considering the activity of immobilization enzyme and cost of lipase and carrier, the concentration of lipase was fixed at 2 mg/mL.

### 3.2. Effect of Temperature and pH on Activity of Free and Immobilized Lipase

**Figure 4:**
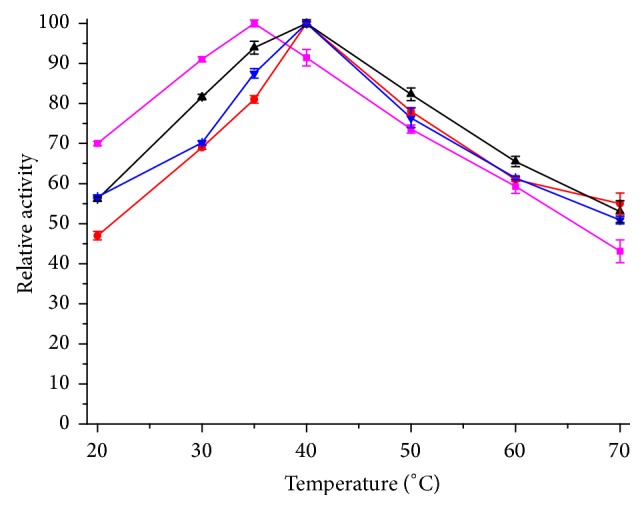
Optimal temperature of immobilized enzyme. This figure shows the relationship between relative activity and temperature. The absolute values of hydrolytic activity: free lipase: 24.3 U/mL; WDA: 350.1 U/g; EP: 240.3 U/g; EA: 169.9 U/g. The color of curves represents the following: magenta: free lipase; red: WDA; black: EP; blue: EA.

The optimal temperature on the activities of the free and immobilized lipase was investigated in the temperature range 20–70°C. The results are given in Figure 4. The optimal temperature of immobilized lipase was 40°C and 5°C higher than free lipase, because thermal stability of lipase was enhanced after immobilization. The activity of immobilized lipase kept more than 50% at 70°C.

**Figure 5:**
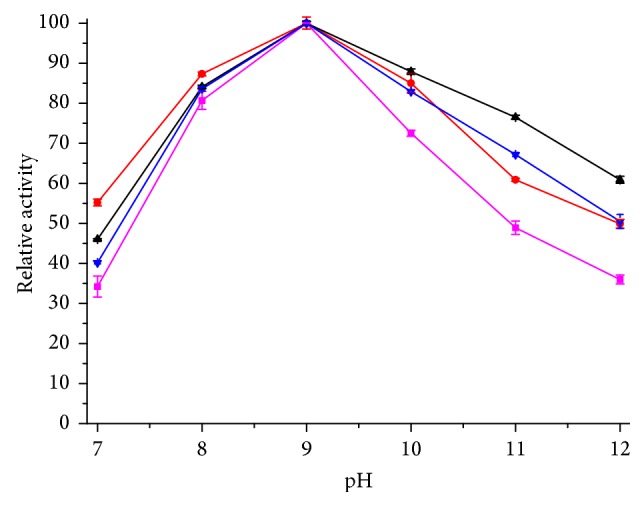
Optimal pH of immobilized enzyme. This figure shows the relationship between relative activity and pH. The absolute values of hydrolytic activity: free lipase: 26.5 U/mL; WDA: 373.2 U/g; EP: 245.7 U/g; EA: 172.6 U/g. The color of curves: magenta: free lipase; red: WDA; black: EP; blue: EA.

The effect of pH on the activity of free and immobilized lipase in* p*-nitrophenyl laurate hydrolysis was determined in the pH range 7–12 and the results are presented in Figure 5. Optimal pH value of free lipase and immobilized lipases was obtained at pH 9. The activity of immobilized lipase was much higher than the free lipase at neutral and alkaline pH especially at alkaline pH (more than 50% at pH 12). Immobilization expanded the application range of lipase to catalyze hydrolysis and esterification in harsh condition.

### 3.3. Temperature and pH Stability of Immobilized Enzyme

**Figure 6:**
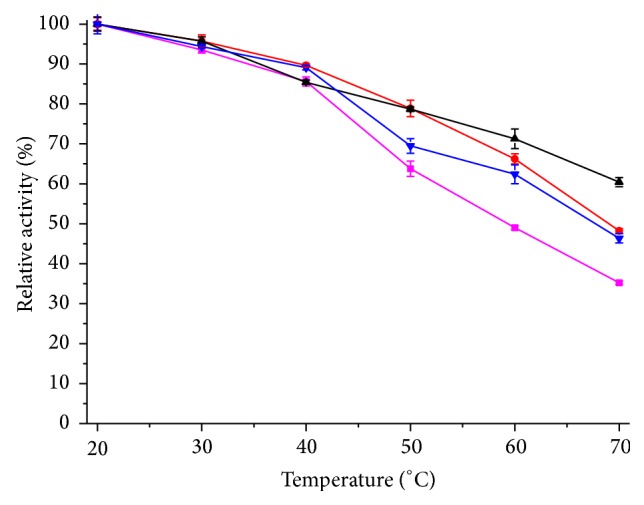
Thermal stability of free and immobilized enzyme. This figure shows the relationship between relative activity and temperature. The absolute values of hydrolytic activity: free lipase: 21.3 U/mL; WDA: 358.5 U/g; EP: 231.4 U/g; EA: 174.2 U/g. The color of curves: magenta: free lipase; red: WDA; black: EP; blue: EA.

The thermal stability on the activities of the free and immobilized lipase was investigated in the temperature range 20–70°C for 3 h. Results shown in Figure 6 indicate that immobilized lipases are much more stable than free lipase at elevated temperatures. At 60°C, free enzyme retained 49% of its initial activity after a 3 h of heat treatment and immobilized lipases kept greater than 62.4% activity. At 70°C, the free enzyme lost most activity (35.3%) and immobilized lipases retained more than 46.4% of their initial activity after a 3 h of heat treatment. Thermal stability of enzyme is greatly enhanced after immobilization, because the carriers may provide an external backbone to keep enzyme's shape which minimizes the negative effect of high temperature.

**Figure 7:**
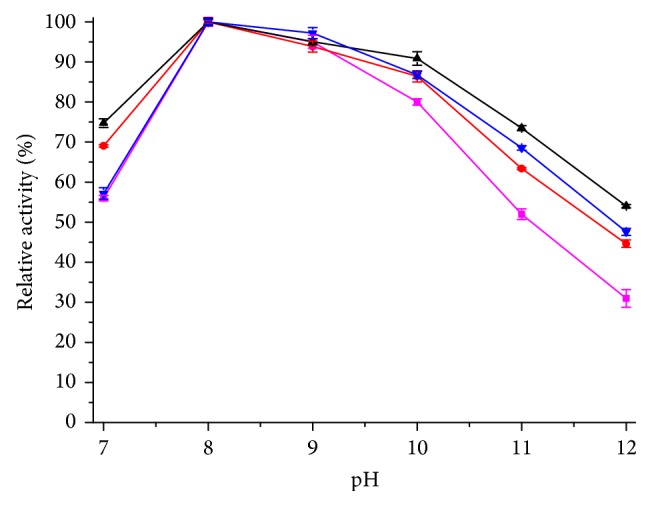
pH stability of free and immobilized enzyme. This figure shows the relationship between relative activity and pH. The absolute values of hydrolytic activity: free lipase: 21.5 U/mL; WDA: 362.1 U/g; EP: 235.7 U/g; EA: 170.3 U/g. The color of curves: magenta: free lipase; red: WDA; black: EP; blue: EA.

pH stability assays of free and immobilized lipases were performed in the pH range 7–12. The obtained results were shown in Figure 7. At alkaline pH, immobilized lipases were more stable than free lipase (residue activity more than 63.4% at pH 11 for 3 h). Immobilization support could maintain the three-dimensional structure of lipase and resist the interference of high pH.

### 3.4. Reusability of Immobilized Lipase

Immobilized lipases retained 80% of their activity after 5 cycles (Table 1). The results mean that the lipase is tightly attached to carriers in the recycling process without obviously losing activity. The residual activity of WDA and EP retained full activity 38.7% and 48.1%, respectively, after 10 cycles. The residual activity of EA retained 52.1% after 10 cycles because of its multipoint covalent attachment. WDA which is immobilized by the absorption method is significantly losing its activity because of the weak interaction. The residual activity of EP which is immobilized by adsorption and covalent attachment was between covalent linkage method and absorption method. Covalent linkage method showed better reusability because the stable chemical bond between enzyme and carrier increased the stability of immobilized enzyme.

### 3.5. Effect of Different Organic Solvents on the Catalytic Activity

The results are presented in Table 2. The activities of immobilized lipase were almost lost in chloroform, n-hexane, and n-heptane. In ethanol and acetone, immobilized lipases could keep more than 47% activity, and the activity of WDA in acetone increased to 106.3%. tert-Butanol could increase the activity of immobilized lipase and the activity of WDA up to 156.6%. In previous works, tert-butanol has been shown to stabilize the activity of* Candida antarctica* lipase [43–45]. tert-Butanol is not a substrate for lipase and is easily removed by washing step. These reasons may cause the enhancement of lipase activity by incubating in tert-butanol. Immobilized lipase catalyzed the interesterification and hydrolysis reaction of unsaturated fatty acid (such as fish oil) and tert-butanol often used as the reaction solvent. These immobilization methods increased or kept the stability of lipase in tert-butanol, ethanol, and acetone. It provides a chance to expand the application of marine lipase in organic system.

## 4. Conclusion

A microbial lipase immobilized by three carriers retained good activity at temperature 40°C and pH 9. Immobilization of lipase expanded the application range of lipase to catalyze hydrolysis and esterification in harsh condition. It improved reusability of lipase and showed enhanced activity when exposed to tert-butanol. It provides a chance to expand the application of marine lipase in organic system.

## Figures and Tables

**Table 1:** Reusability of immobilized lipase.

Cycle	WDA Relative activity (%)	EP Relative activity (%)	EA Relative activity (%)
1	100 (±1.2) (Activity 365.9 U/g)	100 (±0.8) (Activity 238.4 U/g)	100 (±0.5) (Activity 173.8 U/g)
2	92.3 (±0.3)	95.1 (±1)	98.0 (±0.9)
3	86.6 (±0.5)	88.2 (±1.2)	89.7 (±1.5)
4	82.3 (±2.1)	84.9 (±1.8)	89.4 (±1.9)
5	80.1 (±0.8)	80.7 (±0.9)	85.7 (±0.8)
6	69.5 (±1)	73.6 (±0.7)	82.1 (±0.7)
7	60.5 (±0.2)	70.8 (±1.3)	71.5 (±0.1)
8	52.4 (±0.1)	67.8 (±0.6)	63.1 (±1.4)
9	43.9 (±0.9)	53.9 (±0.6)	59.0 (±1.3)
10	38.7 (±0.5)	48.1 (±0.8)	52.1 (±0.2)

**Table 2:** Effect of organic solvents on immobilized lipase activity.

Type	WDA	EP	EA
Solvent	Relative activity (%)	Relative activity (%)	Relative activity (%)
Buffer	100 (±0.3)	100 (±1.4)	100 (±1.2)
Ethanol	77.7 (±0.2)	47.0 (±0.7)	68.7 (±1.2)
tert-Butanol	156.6 (±0.5)	108.7 (±2.4)	114.8 (±2.3)
Acetone	106.3 (±1.5)	66.6 (±1.6)	55.7 (±0.9)
Chloroform	10.2 (±2.1)	14.1 (±0.8)	7.6 (±0.2)
n-Hexane	15.1 (±1)	30.5 (±1.8)	20.0 (±0.8)
n-Heptane	12.1 (±0.6)	24.3 (±1.3)	17.9 (±0.4)
